# LncRNA SNHG6 knockdown inhibits cisplatin resistance and progression of gastric cancer through miR-1297/BCL-2 axis

**DOI:** 10.1042/BSR20211885

**Published:** 2021-12-08

**Authors:** Jiazhuan Mei, Guiju Liu, Ruijun Li, Peng Xiao, Dan Yang, Hua Bai, Yibin Hao

**Affiliations:** Department of Oncology, People’s Hospital of Zhengzhou Affiliated to Southern Medical University, Zhengzhou, Henan, China

**Keywords:** BCL-2, cisplatin resistance, Gastric cancer, lncRNA SNHG6, miR-1297

## Abstract

Cisplatin (DDP) resistance is a huge obstacle to gastric cancer (GC) treatment. Long non-coding RNAs (lncRNAs) have been manifested to exert pivotal functions in GC development. Herein, we aimed to explore the functional impact of lncRNA small nucleolar RNA host gene 6 (SNHG6) on DDP resistance and progression of GC. Quantitative real-time PCR (qRT-PCR) assay or Western blotting was performed to detect the expression of SNHG6, microRNA(miR)-1297, and epithelial–mesenchymal transition (EMT)-related factors and B-Cell Lymphoma 2 (Bcl-2) in DDP-resistant GC cells. Half inhibition concentration (IC50) to DDP, clonogenicity, apoptosis and invasion were examined via CCK-8 assay, colony formation assay, flow cytometry and Transwell assay, respectively. Target association between miR-1297 and SNHG6 or BCL-2 was demonstrated via dual-luciferase reporter assay or RIP assay. Xenograft models in nude mice were formed to investigate role of SNHG6 *in vivo*. We found that SNHG6 and BCL-2 were up-regulated, while miR-1297 expression was declined in GC tissues and DDP-resistant cells. Moreover, depletion of SNHG6 or gain of miR-1297 could repress DDP resistance, proliferation and metastasis of DDP-resistant cells, which was weakened by miR-1297 inhibition or BCL-2 overexpression. Besides, SNHG6 positively regulated BCL-2 expression by sponging miR-1297. Furthermore, SNHG6 knockdown repressed GC tumor growth *in vivo*. In a word, lncRNA SNHG6 knockdown had inhibitory effects on DDP resistance and progression of GC by sponging miR-1297, highlighting its potential in GC treatment.

## Introduction

Gastric cancer (GC) is a most common tumor around the world, serving as third contributor of malignancy-associated deaths [1]. Although many novel approaches have applied to GC treatment [2], the 5-year survival remains very poor due to lack of biomarker and cell resistance to chemotherapy agents, such as cisplatin (DDP) [3,4]. Therefore, deeper exploration on the mechanistic pathway of GC tumorigenesis and DDP resistance is greatly significant.

Long non-coding RNAs (lncRNAs) are a category of non-protein-coding RNAs with lengths exceeding 200 nucleotides (nts) and act as important regulators in cancer development owing to their specific roles in tumorigenesis and metastasis by mediating gene transcription and post-transcriptional modification. For instance, lncRNAs can bind with the promoter region of hTERT to regulate target gene expression. LncRNAs mediate mRNA degradation to change malignant phenotypes [5,6]. Many lncRNAs have been certified to be concerned with the cancer chemoresistance, including GC [7,8]. LncRNAs are also key controllers in initiation and progression of GC [9]. Small nucleolar RNA host gene 6 (SNHG6) is deemed as a promising prognostic biomarker of many human cancers and has close association with several clinicopathological characteristics [10]. SNHG6, an oncogene in GC, is demonstrated to be a potent prognostic biomarker and a therapy target for GC [11]. However, the molecular mechanism of SNHG6 in GC progression requires further research.

MicroRNAs (miRNAs) are small, endogenous noncoding RNAs with lengths 22 nts, which affect animal development and disease [12]. miRNAs could function as promising biomarkers of GC chemoresistance and are involved in regulation of chemo-sensitivity and chemo-resistance during GC progression [13]. miR-1297 could exert tumor-suppressing role in several malignancies, such as lung cancer, pancreatic cancer, hepatocellular carcinoma and colorectal cancer [14–17], or act as an oncogene in NSCLC, liver cancer, LSCC and cervical carcinoma [18–21]. In GC, miR-1297 expression is greatly declined in GC, and it might exert a significant role in GC development [22]. However, the involvement of miR-1297 in DDP resistance of GC remains to be substantiated.

B-cell lymphoma 2 (BCL-2) is an important inhibitor of cell death, associated with a complex network to regulate apoptosis [23]. Inhibition of BCL-2 could relieve apoptosis of tumor cells or make them more sensitive to chemotherapy or radiotherapy. Thus, BCL-2 is regarded as a target of anti-cancer agents [24]. BCL-2 is involved in miR-136-induced apoptosis of GC cells [25]. Additionally, up-regulation of BCL-2 could reduce efficacy of several GC chemotherapy agents [26]. As a promising candidate of miR-1297, the co-effect of BCL-2 and miR-1297 on DDP resistance of GC is valuable to be clarified.

In this project, the dysregulation of lncRNA SNHG6 in GC tissues and DDP-resistant cells was detected. Moreover, functional impact of SNHG6 on DDP resistance and progression of GC, as well as the possible mechanistic pathway were investigated.

## Materials and methods

### Patients and tissue collection

Before implementing this project, we got the authorization from the Ethics Committee of People’s Hospital of Zhengzhou. Thirty-five GC tissues and paired normal tissues were excised from GC patients enrolled at People’s Hospital of Zhengzhou (Supplementary Table S1). All these individuals supplied written informed consents prior to surgery, and none of them accepted any therapeutic approaches.

### Cell culture

Human normal gastric epithelial cell line GES-1 (CL-0563; Procell, Wuhan, China) and GC cell line MKN-45 (CL-0292; Procell), AGS (CRL-1739; ATCC, Manassas, VA, U.S.A.) were maintained in RPMI-1640 medium (Gibco, Grand Island, NY, U.S.A.) mixed with 10% FBS (Gibco) and 1% penicillin/streptomycin (Sigma-Aldrich, St. Louis, MO, U.S.A.) at 37°C with 5% CO_2_. Additionally, 293T cells (CRL-11268; ATCC) were maintained in DMEM (30-2002, ATCC).

GC cell line with cisplatin (DDP) resistance was established referring to a former work [27]. DDP concentration in RPMI-1640 medium for cell culture was gradually increased. 6 months later, DDP concentration in medium was altered to 2 µg/ml to maintain resistance.

### Cell transfection

To silence lncRNA SNHG6, small interfering RNA (si-lnc SNHG6) and negative control (si-NC) were constructed by GENEWIZ (Suzhou, China). miR-1297 mimic (miR-1297), miR-1297 inhibitor (anti-miR-1297) and control (miR-NC and anti-NC) were supplied by RIBOBIO Co. Ltd. (Guangzhou, China). To overexpress BCL-2, its full-length cDNA sequence was inserted into pcDNA 3.1 vector (Addgene, Cambridge, MA, U.S.A.) to synthesize pcDNA 3.1-BCL-2 (BCL-2) and the empty vector was regarded as negative control. Aforementioned oligonucleotides or plasmids were transfected into AGS/DDP and MKN-45/DDP cells exploiting Lipofectamine 3000 (Thermo Fisher Scientific, Waltham, MA, U.S.A.) in conformity to the user’s manual.

### Quantitative real-time PCR (qRT-PCR)

Clinical tissues and cultured cells were subjected to RNA extraction utilizing TRIzol Reagent (Beyotime, Shanghai, China). Afterwards, 1 μg RNA was used for cDNA generation with M-MLV reverse transcriptase (Beyotime) or TaqMan reverse transcription kit (Thermo Fisher Scientific). Following qRT-PCR was performed with SYBR Green Master Mix (TaKaRa, Otsu, Japan) or TaqMan MicroRNA Assays (Thermo Fisher Scientific). Relative expression was analyzed using 2^−ΔΔCt^ quantification method, normalized to U6 (for miR-1297) or glyceraldehyde-3-phosphate dehydrogenase (GAPDH, for other genes). The sequence of all primers of qRT-PCR assay were as followed: lncRNA SNHG6-forward (F): 5′-ATACTTCTGCTTCGTTACCT-3′ and lncRNA SNHG6-reverse (R): 5′-CTCATTTTCATCATTTGCT-3′; GAPDH-F: 5′-AATCCCATCACCATCTTCC-3′ and GAPDH-R: 5′-CATCACGCCACAGTTTCC-3′; miR-1297-F: 5′-ACACTCCAGCTGGGTTCAAGUAATT-3′ and miR-1297-R: 5′-TGGTGTCGTGGAGTCG-3′; U6-F: 5′-CTCGCTTCGGCAGCACA-3′ and U6-R: 5′-AACGCTTCACGAATTTGCGT-3′; E-cadherin-F: 5′-GTCAGTTCAGACTCCAGCCC-3′ and E-cadherin-R: 5′-AAATTCACTCTGCCCAGGACG-3′; N-cadherin-F: 5′-GGACAGCCTCTTCTCAATG-3′ and N-cadherin-R: 5′-CTGCAGGCTCACTGCTCTC-3′; Vimentin-F: 5′-AAAGTGTGGCTGCCAAGAAC-3′ and Vimentin-R: 5′-AGCCTCAGAGAGGTCAGCAA-3′.

### CCK-8 assay

The current assay was hired to examine the DDP resistance of GC cells. Cells (∼3 × 10^3^) were inoculated in 96-well plates, followed by DDP treatment. Then, 10 µl CCK-8 solution (Beyotime) was pipetted into every well. Two hours later, the absorbance at 450 nm of was checked utilizing a microplate reader, and then the IC50 value was analyzed.

### Cell colony formation assay

Transfected AGS/DDP and MKN-45/DDP cells (∼1000 cells/well) were plated into 6-well plates. Following 10-day routine culture, formed cell colonies were subjected to immobilization with methanol and dying with Crystal Violet (Beyotime). Later, colonies were counted utilizing ImageJ software (NIH, Bethesda, MD, U.S.A.). The colony formation efficiency indicates the percentage of the number of colonies among number of inoculated cells.

### Flow cytometry

For cell apoptosis analysis, the present assay was executed with Annexin V-FITC Apoptosis Detection Kit (Beyotime) referring to the user’s manual. AGS/DDP and MKN-45/DDP cells were harvested after transfection. Following re-suspension into single cell, Annexin V-FITC and PI were pipetted to dye the cells in darkness. Subsequently, apoptotic cells (Annexin V +) were examined exploiting flow cytometer (BD Biosciences, San Jose, CA, U.S.A.).

### Transwell invasion assay

Cell invasion was assessed utilizing Transwell chamber (8 µm size, Millipore, Billerica, MA, U.S.A.) adhered with Matrigel (BD Biosciences). A total of 1 × 10^5^ transfected AGS/DDP and MKN-45/DDP cells were put into the insert chamber containing non-serum RPMI-1640, while complete RPMI-1640 was added in the bottom chamber as nutrient. After 24 h, cells invaded to the other side of the inserts were immobilized and dyed for photographing and counting under a microscope (100×). Noteworthily, the number of invaded cells represents the mean value of cells in five random fields.

### Dual-luciferase reporter assay (DLRA)

The targets of SNHG6 and miR-1297 were estimated by Starbase 3.0, and miR-1297 was found to bind with SNHG6 and 3′ untranslated region (3′UTR) of BCL-2. The partial sequence of SNHG6 or BCL-2 3′UTR harboring binding position with miR-1297 was inserted into pMirGLO reporter vector (Promega, Shanghai, China) to create wild-type luciferase reporter plasmid SNHG6-WT or 3′UTR of BCL-2-WT. The mutant type reporters were synthesized via inserting mutant binding position. Later, each construct and miR-NC or miR-1297 were co-introduced into 293T cells utilizing Lipofectamine 3000. Forty-eight hours later, luciferase density was detected with Dual-Glo Luciferase Assay System kit (Promega).

### RIP assay

An EZ-Magna RIP Kit (Millipore) was applied for this assay according to the guideline of manufacturer. AGS/DDP and MKN-45/DDP cells were harvested and lysed in lysis buffer. Obtained cell lysate was mixed with RIP buffer embracing magnetic beads which were conjugated with anti-Ago2 or anti-IgG antibody, and IgG or Input was acted as control. After overnight incubation, beads were treated with Proteinase K to extract immunoprecipitated RNA. Isolated RNA was subjected for qRT-PCR assay to analyze the abundance of SNHG6 and miR-1297.

### Western blot assay

Protein samples were extracted from clinical tissues and cultured cells utilizing RIPA buffer (Beyotime). After quantification using a BCA Kit (Sigma-Aldrich), 40 μg samples were segregated and eletro-transferred onto PVDF membranes (Millipore). After blockage in 5% fat-free milk, these membranes were incubated with the primary antibody against BCL-2 (ab182858; Abcam, Shanghai, China; 1:1000 dilution), against cleaved-Caspase3 (ab32042; Abcam, Shanghai, China; 1:1000 dilution), against pro-Caspase3 (ab32351; Abcam, Shanghai, China; 1:1000 dilution) or GAPDH (ab181602; Abcam; 1:2000 dilution), then reacted with secondary antibody (ab205718; Abcam; 1:5000 dilution). For visualization of protein bands, ECL western blot detection system (Millipore) was applied. Later, the relative expression of BCL-2, cleaved-Caspase3 or pro-Caspase3 were analyzed by normalization to GAPDH.

### Tumor xenograft assay

The present assay operated on mice were approved by the Ethics Committee of People’s Hospital of Zhengzhou, and all mice experiments took place at central laboratory of People’s Hospital of Zhengzhou. Five-week-old nude mice were procured from Beijing Laboratory Animal Center (Beijing, China). ∼ 2 × 10^6^ AGS/DDP cells stably introduced with lentiviral vector silencing SNHG6 (sh-SNHG6; HanBio, Shanghai, China) or blank control (sh-NC; HanBio) were subjected for hypodermic injection in the right flank of the nude mice. One week later, the mice were subjected to intraperitoneal injection with DDP diluted in PBS (5 mg/kg) or only PBS 3 times per week (*n*=5). The size of tumors was measured once a week and computed exploiting the following formula: 0.5 × length × width^2^. After additional 4 weeks, all animals were euthanized with inhalation anesthesia of 5% isoflurane and cervical dislocation, and formed tumors were resected for weigh.

### Statistical analysis

All data were generated from exceeding three biological repetitions then processed using SPSS 21.0. Data were expressed as mean ± SD. Difference analysis was determined via Student’s *t*-test or ANOVA. Correlation between expression of SNHG6 and miR-1297 in GC tissues was determined via Spearman’s correlation analysis. When *P*-value < 0.05, it was deemed to be significant.

## Results

### Determination of lncRNA SNHG6 expression in GC tissues and cells

**Figure 1:**
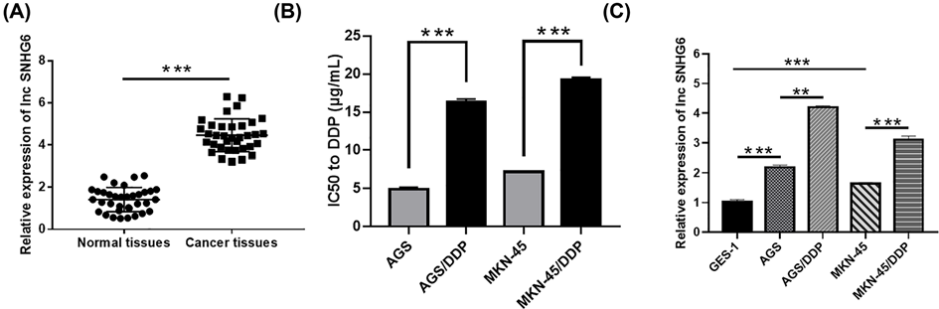
Determination of lncRNA SNHG6 expression in GC tissues and cells (**A**) qRT-PCR assay for the abundance of SNHG6 in 35 GC tissues and paired normal tissues. (**B**) CCK-8 assay for the IC50 of AGS, AGS/DDP, MKN-45 and MKN-45/DDP cells to DDP. (**C**) qRT-PCR assay for the expression of SNHG6 in GES-1, AGS, AGS/DDP, MKN-45 and MKN-45/DDP cells; **P<0.01; ***P<0.001.

qRT-PCR assay presented that SNHG6 expression was apparently elevated in GC tissues in reference to normal tissues (Figure 1A). CCK-8 assay revealed that IC50 of AGS/DDP and MKN-45/DDP cells to DDP was higher than that of AGS and MKN-45 cells, respectively, indicating the successful establishment of the two DDP-resistant cell lines (Figure 1B). The obvious upregulation of SNHG6 was also discovered in GC cells in comparison with GES-1 cells, especially in DDP-resistant cells (AGS/DDP and MKN-45/DDP) compared with their parental DDP-sensitive cells (Figure 1C). Collectively, SNHG6 might exert tumor-promoting role in GC.

### Role of SNHG6 in DDP resistance, proliferation and metastasis of DDP-resistant GC cells

**Figure 2:**
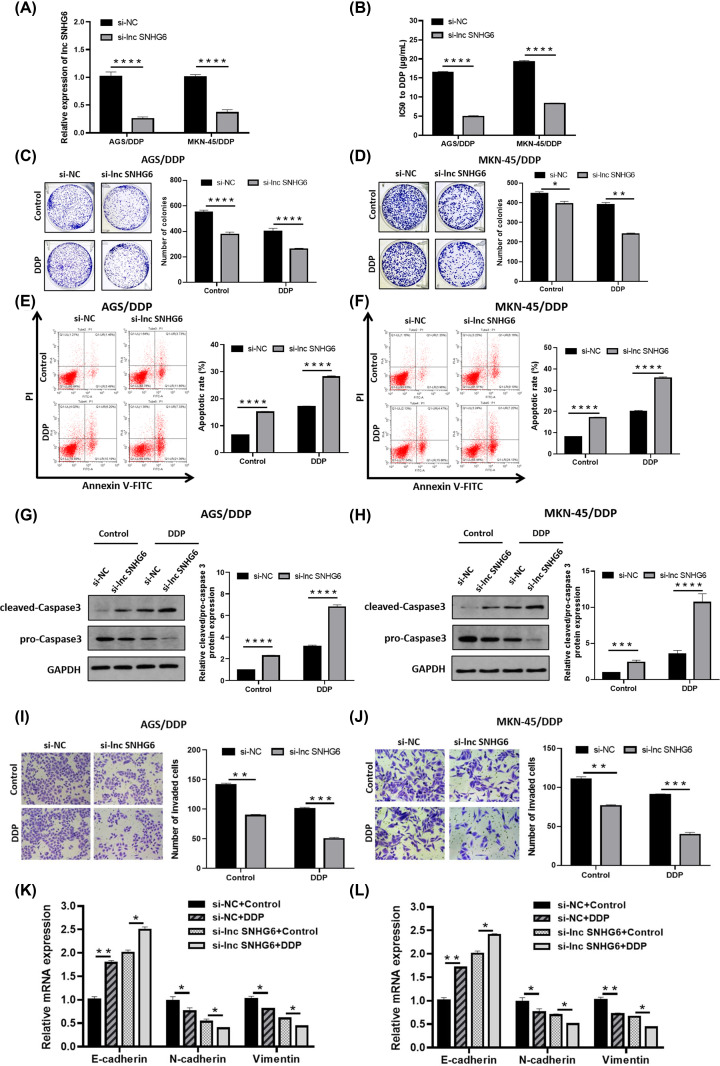
Role of SNHG6 in DDP resistance, proliferation and metastasis of DDP-resistant GC cells (**A**) qRT-PCR assay for the enrichment of SNHG6 in AGS/DDP and MKN-45/DDP cells introduced with si-lnc SNHG6 or si-NC. (**B**) CCK-8 assay for the IC50 of AGS/DDP and MKN-45/DDP cells introduced with si-lnc SNHG6 or si-NC to DDP. (**C–J**) AGS/DDP and MKN-45/DDP cells introduced with si-lnc SNHG6 or si-NC were disposed with DDP (final concentration: 15 µg/ml) or Control (isopyknic PBS solution). Cell clonogenicity (C,D), apoptosis (E–H) invaded potency (I and J) and E-cadherin, N-cadherin and Vimentin mRNA expression were analyzed (**K** and **L**); *P<0.05; **P<0.001; ***P<0.001; ****P<0.0001.

Having known the dysregulation of SNHG6 in GC tissues, we then studied the function of SNHG6 by loss-of-function assay. First of all, SNHG6 in AGS/DDP and MKN-45/DDP cells was knocked down via transfecting siRNA targeting SNHG6 and si-NC. The knockdown efficiency was exhibited in Figure 2A, as demonstrated by qRT-PCR assay. CCK-8 assay displayed that depletion of SNHG6 apparently reduced the IC50 of AGS/DDP and MKN-45/DDP cells after treating with DDP (Figure 2B). SNHG6 knockdown also reduced the clonogenicity of DDP-resistant cells treated with DDP or not (Figure 2C,D). Data from flow cytometry highlighted the SNHG6 knockdown-induced cell apoptosis were increased in AGS/DDP and MKN-45/DDP cells (Figure 2E,F). Moreover, the apoptotic-related protein cleaved-Caspase3 was enhanced, and pro-Caspase3 level was inhibited in AGS/DDP and MKN-45/DDP cells (Figure 2G,H). For cell metastasis analysis, we found that SNHG6 deficiency effectively suppressed cell invasion and EMT process in DDP-resistant cells, which were manifested by declined number of invaded cells, increased level of E-cadherin and declined levels of N-cadherin and Vimentin (Figure 2I–L), implying that SNHG6 depletion block DDP-resistant cell metastasis. Taken together, SNHG6 knockdown repressed DDP resistance, proliferation and metastasis of DDP-resistant GC cells.

### Identification of miR-1297 as a target of SNHG6

**Figure 3:**
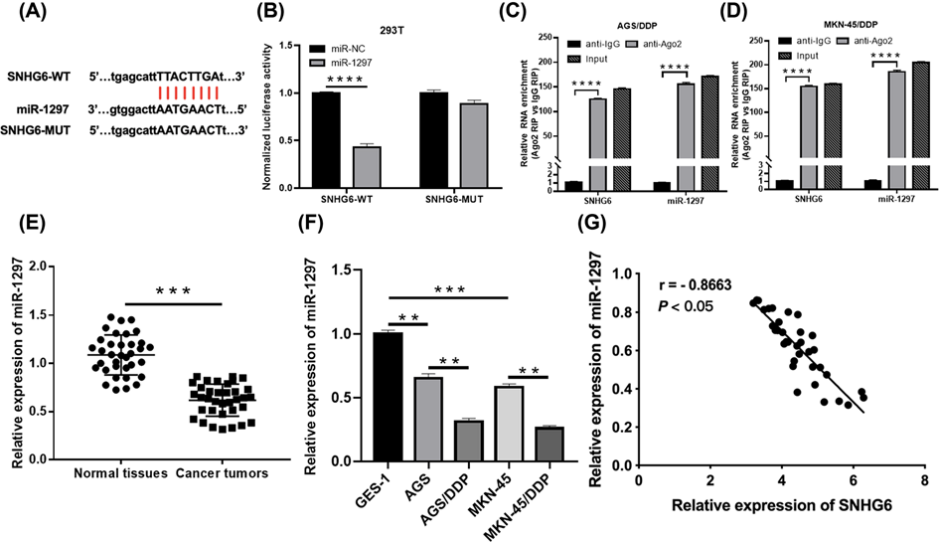
Identification of miR-1297 as a target of SNHG6 (**A**) Binding position of SNHG6 and miR-1297. (**B**) DLRA for the luciferase density of SNHG6-WT and SNHG6-MUT. (**C** and **D**) RIP assay for the potential endogenous combination of SNHG6 and miR-1297. (**E**) qRT-PCR assay for the enrichment of miR-1297 in 35 GC tissues and paired normal tissues. (**F**) qRT-PCR assay for the enrichment of miR-1297 in GES-1, AGS, AGS/DDP, MKN-45 and MKN-45/DDP cells. (**G**) Spearman’s correlation analysis for expression of SNHG6 and miR-1297 in 35 GC tissues; **P<0.001; ***P<0.001; ****P<0.0001.

LncRNAs are proved to exert regulatory roles in GC by serving as ceRNAs to interact with miRNAs [28]. Here, we searched the target miRNA of SNHG6 for clarifying the mechanism. As forecasted by Starbase 3.0, SNHG6 had binding sites with miR-1297 (Figure 3A). Gain of miR-1297 obviously reduced the luciferase density of SNHG6-WT, while it had no remarkable influence on that of SNHG6-MUT (Figure 3B). RIP assay testified that SNHG6 and miR-1297 were highly enriched by anti-Ago2 in contrast with anti-IgG, suggesting the endogenous combination between SNHG6 and miR-1297 (Figure 3C,D). Afterwards, we found the down-regulation of miR-1297 in GC tissues and GC cells, especially in AGS/DDP and MKN-45/DDP cells (Figure 3E,F). There was a negative correlation between expression of miR-1297 and SNHG6 in GC tissues (Figure 3G). In sum, SNHG6 could target miR-1297 in GC.

### Effect of miR-1297 on DDP resistance, proliferation and metastasis of DDP-resistant GC cells with SNHG6 knockdown

**Figure 4:**
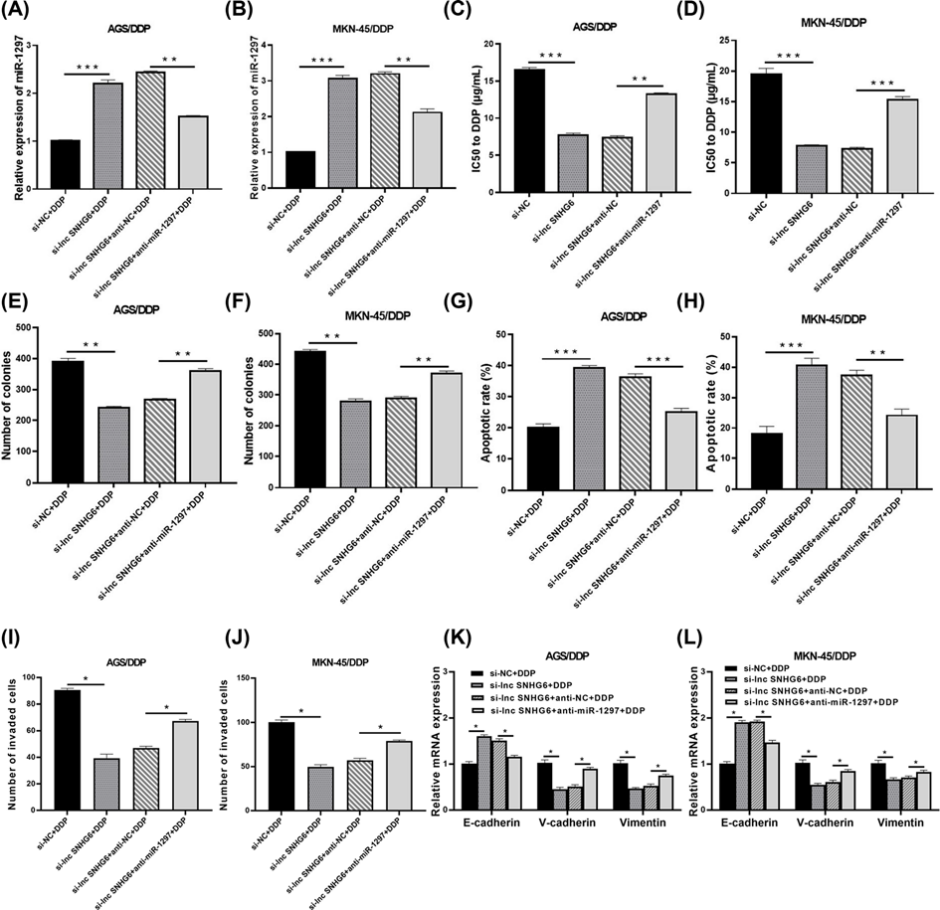
Effect of miR-1297 on DDP resistance, proliferation and metastasis of DDP-resistant GC cells with SNHG6 knockdown AGS/DDP and MKN-45/DDP cells introduced with si-lnc SNHG6, si-lnc SNHG6+anti-NC, si-lnc SNHG6+anti-miR-1297 or si-NC, were disposed with DDP (Final concentration: 15 µg/mL). (**A** and **B**) qRT-PCR assay for abundance of miR-1297. (**C–L**) IC50 (C,D), clonogenicity (E,F), apoptosis (G,H), invaded potency (I,J), and E-cadherin, N-cadherin and Vimentin mRNA expression (K,L) were examined; *P<0.05; **P<0.001; ***P<0.001.

Then the co-effect of SNHG6 and miR-1297 on the resistance, proliferation and metastasis of DDP-resistant GC cells was studied. As depicted in Figure 4A,B, SNHG6 knockdown elevated the enrichment of miR-1297 in AGS/DDP and MKN-45/DDP cells, which was weakened by interference of miR-1297. Furthermore, SNHG6 knockdown-induced biological function, including IC50 to DDP (Figure 4C,D), clonogenicity (Figure 4E,F), cell apoptosis (Figure 4G,H) and cell metastasis (Figure 4I–L) in DDP-resistant GC cells, were all attenuated by additional miR-1297 inhibitor. Above results implied that SNHG6 knockdown inhibited DDP resistance, proliferation and metastasis of DDP-resistant GC cells by increasing miR-1297 expression.

### Identification of BCL-2 as a target of miR-1297

**Figure 5:**
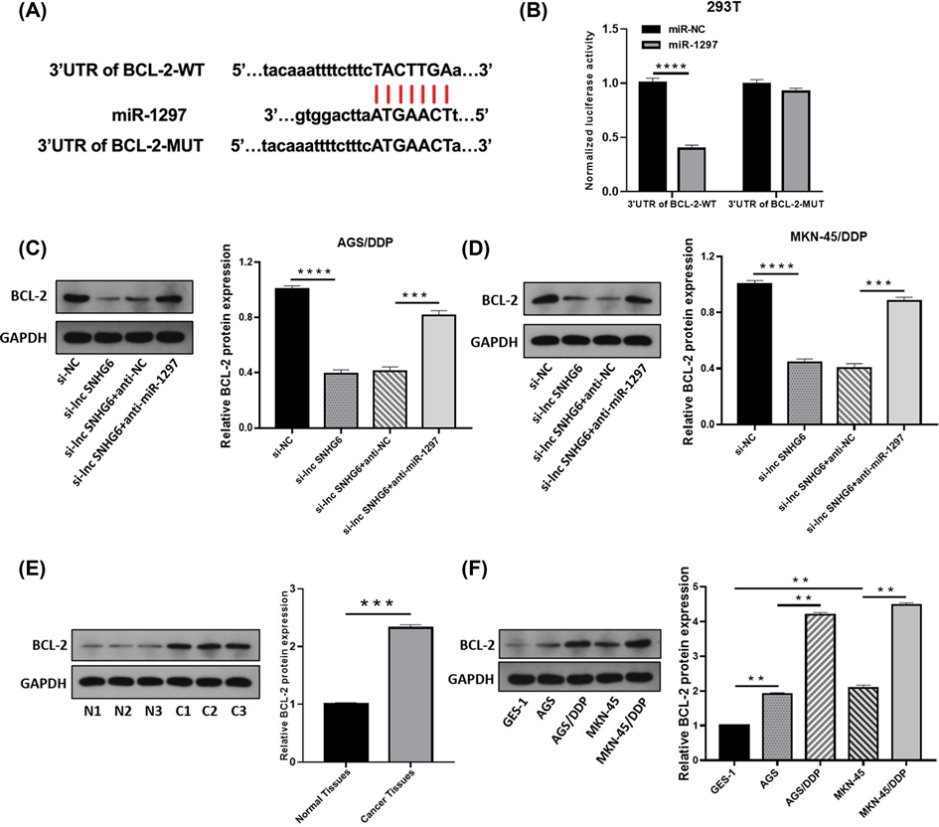
Identification of BCL-2 as a target of miR-1297 (**A**) Binding region of 3′UTR of BCL-2 and miR-1297. (**B**) DLRA for the luciferase density of 3′UTR of BCL-2-WT and 3′UTR of BCL-2-MUT. (**C–F**) Western blotting for the abundance of BCL-2 protein in AGS/DDP and MKN-45/DDP cells introduced with si-lnc SNHG6, si-lnc SNHG6+anti-NC, si-lnc SNHG6+anti-miR-1297 or si-NC (C and D), in 35 GC tissues and paired normal tissues (E), or in GES-1, AGS, AGS/DDP, MKN-45 and MKN-45/DDP cells (F); **P<0.001; ***P<0.001; ****P<0.0001.

Starbase 3.0 was also utilized to estimate the target gene of miR-1297, and 3′UTR of BCL-2 was discovered to endow with the complementary region with miR-1297 (Figure 5A). DLRA witnessed the miR-1297-induced the reduction of the luciferase activity of 3′UTR of BCL-2-WT in 293T cells, but that of BCL-2-MUT was changeless, suggesting the target relationship between miR-1297 and BCL-2 (Figure 5B). As exhibited in Figure 5C,D, SNHG6 knockdown strikingly reduced protein level of BCL-2, which was reversed by miR-1297 inhibition. In addition, BCL-2 was up-regulated in GC tissues (Figure 5E) and DDP-resistant GC cells (Figure 5F) when compared with respective controls. Therefore, miR-1297 could target BCL-2.

### Co-effect of miR-1297 and BCL-2 on DDP resistance, proliferation and metastasis of DDP-resistant GC cells

**Figure 6:**
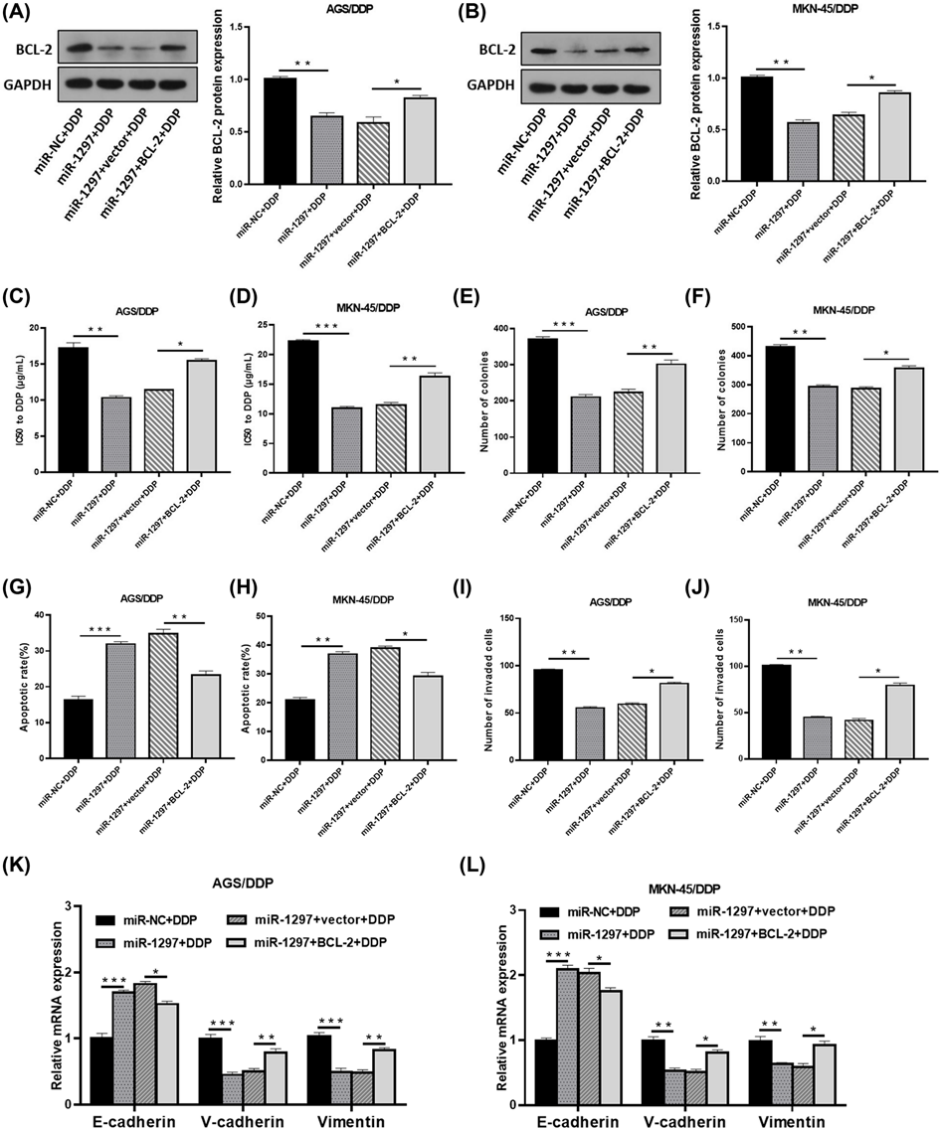
Co-effect of miR-1297 and BCL-2 on DDP resistance, proliferation and metastasis of DDP-resistant GC cells AGS/DDP and MKN-45/DDP cells introduced with miR-1297, miR-1297+vector, miR-1297+BCL-2 or miR-NC were disposed with DDP (final concentration: 15 µg/ml). (**A** and **B**) Western blotting for the abundance of BCL-2 protein. (**C–L**) IC50 (C and D), clonogenicity (E and F), apoptosis (G and H), invaded potency (I and J) and E-cadherin, N-cadherin and Vimentin mRNA expression (K and L) were assessed; *P<0.05; **P<0.001; ***P<0.001.

Transfection of miR-1297 strikingly decreased the abundance of BCL-2 protein in AGS/DDP and MKN-45/DDP cells, while overexpressed BCL-2 relieved the inhibitory effect (Figure 6A,B). Gain of miR-1297 also reduced the IC50 of DDP-resistant GC cells to DDP (Figure 6C,D). Enforced expression of miR-1297 caused the declined clonogenicity (Figure 6E,F), increased cell apoptosis (Figure 6G,H) and inhibited cell metastasis (Figure 6I–L), which were largely recovered due to overexpression of BCL-2. Taken together, miR-1297 could suppress the DDP resistance, proliferation and metastasis of DDP-resistant GC cells by directly targeting BCL-2.

### Role of SNHG6 in GC tumor growth

**Figure 7:**
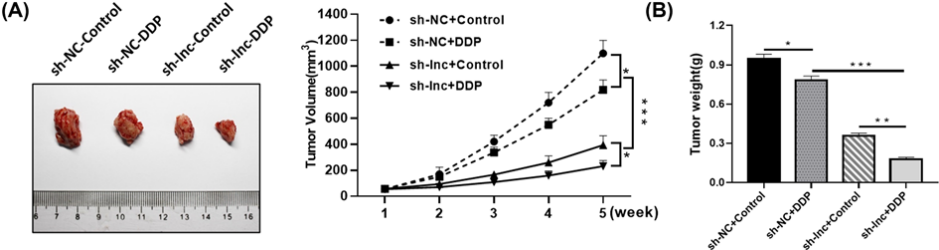
Role of SNHG6 in GC tumor growth (**A**) The volume of generated tumors measured once a week. (**B**) The weight of generated tumors; *P<0.05; **P<0.001; ***P<0.001.

In an attempt to explore the functional role of lnc SNHG6 *in vivo*, the mice were hypodermically injected with AGS/DDP cells expressing sh-lnc or sh-NC, followed by intraperitoneal injection with DDP or PBS. As exhibited in Figure 7A,B, DDP disposition or SNHG6 knockdown obviously inhibited the volume and weight of generated tumors in contrast with corresponding control. Collectively, SNHG6 deficiency hindered GC tumor growth *in vivo*.

## Discussion

Previous works have proved that lncRNAs are implicated with drug resistance of human malignancies, including GC [29]. For example, lncRNA MALAT1 could decrease GC cell resistance chemotherapeutic drugs through miR-23b-3p/ATG12 axis [30]. Silencing of lncRNA PVT1 inhibited DDP resistance in GC via absorbing miR-3619-5p and reducing TBL1XR1 expression [31]. Additionally, lncRNA PCAT-1 could potentiate DDP resistance of GC cells by miR-128/ZEB1 axis [32]. In this project, abundance of lnc SNHG6 was raised in GC tissues and DDP-resistant cells. And silenced SNHG6 reduced DDP resistance and progression of GC, offering new ideas on the molecular mechanism of chemoresistance.

Yan et al. alleges that SNHG6 expression is increased in GC issues and cells and SNHG6 facilitates GC progression through miR-101-3p/ZEB1 axis and epigenetic silencing of p27 [11]. Moreover, another study corroborates that enrichment of SNHG6 is enhanced and interference of SNHG6 impedes GC cell proliferation and tumorigenesis, and triggered cellular senescence by upregulating p21 [33]. Likewise, we also found the augment of SNHG6 expression in GC tissue samples and DDP-resistant cells, as well as the silenced SNHG6 could reduce DDP-resistance, inhibit proliferation and metastasis in DDP-resistant cells by regulating EMT-related proteins expression, indicating SNHG6 plays a vital role in regulating the progression of DDP-resistant cells.

As we all know, lncRNA could interact with miRNAs to participate in biological processes during tumor development [34]. Hence, we tried to search target miRNA of SNHG6. Subsequently, miR-1297 was estimated to be a candidate, as demonstrated by dual-luciferase reporter and RIP assays. miR-1297 could exert cancer-suppressing role in GC, in part, by downregulating CREB1 or CDC6 [22,35]. Additionally, miR-1297 participates in GC tumorigenesis and progression through MALAT1/miR-1297/HMGB2 axis [36] or HOXA11-AS/miR-1297/EZH2 pathway [37]. Although the SNHG6/miR-1297 pathway has been manifested to regulate genome-wide hypomethylation in hepatoma cells [38], the involvement of SNHG6/miR-1297 axis in DDP-resistance of GC is poorly known. In the present study, our data showed lower expression of miR-1297 was observed in GC tissues and cells, as well as a negative correlation was found between SNHG6 and miR-1297. In addition, interference of miR-1297 largely attenuated SNHG6 knockdown-induced damage on DDP resistance, proliferation and metastasis of DDP-resistant cells. In other words, SNHG6 knockdown suppressed progression of DDP-resistant cells by up-regulating miR-1297.

LncRNA/miRNA/mRNA axis has been disclosed to confer important regulation on tumor growth, aggressiveness and metastasis [39]. BCL-2 was validated to be a downstream gene of miR-1297 in this project. Not only involved in GC progression, precious research uncovered that BCL-2 also takes part in regulation on resistance of GC cells [25,40–44]. Here, the up-regulation of BCL-2 was discovered in GC tissues and DDP-resistant cells. Moreover, our data indicated that miR-1297 exerted its regulatory effect on proliferation, metastasis and apoptosis of GC cell by BCL-2. Moreover, overexpression of BCL-2 abated miR-1297-mediated inhibited progression of DDP-resistant cells. We monitored a positive correlation between BCL-2 and E-cadherin expression and an inverse correlation between BCL-2 and N-cadherin and vimentin expression. Thus, SNHG6 affected DDP resistance and progression of GC via modulating miR-1297/BCL-2 axis.

In conclusion, we corroborated that SNHG6 level was greatly enriched in GC tissues and DDP-resistant cells relative to matched controls. Moreover, our data showed that depletion of SNHG6 could reduce DDP resistance and development of GC *in vitro* and *in vivo*. Mechanistically, SNHG6 might function by sponging miR-1297 to activate the expression of BCL-2. Collectively, our study highlighted a novel molecular mechanistic way of the DDP resistance in GC.

## Supplementary Material

Supplementary Table S1

Supplementary Data

## Data Availability

The data and material presented in this manuscript is available from the corresponding author on reasonable request.

## Abbreviations

Bcl-2: B-cell lymphoma 2
DDP: cisplatin
EMT: epithelial–mesenchymal transition
GC: gastric cancer
lncRNA: long non-coding RNA
qRT-PCR: quantitative real-time PCR
SNHG6: small nucleolar RNA host gene 6
