# Supplementary material for: LncRNA SNHG6 knockdown inhibits cisplatin resistance and progression of gastric cancer through miR-1297/BCL-2 axis
Source: Biosci Rep. 2021 Dec 8;41(12):BSR20211885. doi: 10.1042/BSR20211885 (PMC8661508; doi:10.1042/BSR20211885)
Supplement: Supplementary Table S1 [file BSR-2021-1885_supp.pdf]

Supplementary Table 1 Epidemiological data

**No**	**Gender**	**Age**	**Clinical Stages**
**1**	Male	69	T2N0M0
**2**	Female	56	cT4bN2bm0, IIIC
**3**	Male	63	T1N0M0
**4**	Male	56	pT2N1M0
**5**	Female	71	T1N0M0
**6**	Female	69	T2N1M0
**7**	Male	69	pT3N0M0
**8**	Male	53	T2N0M1
**9**	Male	38	T1N0M0
**10**	Female	58	pT2N0M0
**11**	Male	49	pT3N0M0, IIA
**12**	Male	61	T2N0M4
**13**	Female	58	T2N0M2
**14**	Male	70	T1N0M0
**15**	Female	52	T1N0M0
**16**	Female	48	pT4bN1cM0, IIIC
**17**	Female	67	T3N4M1
**18**	Female	56	pT4bN0M0, IIC
**19**	Male	72	T1N0M1
**20**	Male	60	T1N0M0
**21**	Male	44	pT2N0M0 I
**22**	Male	54	T1N0M0
**23**	Female	65	pT4N1M0
**24**	Female	61	T1N0M0
**25**	Female	56	T2N1M4
**26**	Female	74	T1N0M0
**27**	Female	63	T2N0M2
**28**	Male	52	T1N0M0
**29**	Male	66	pT4N0M0
**30**	Female	67	T2N0M0
**31**	Male	63	T2N0M1
**32**	Male	63	T1N0M2
**33**	Male	55	T1N0M0
**34**	Male	80	T1N0M0
**35**	Male	64	pT3N1M0, IIIB
