# Supplementary material for: LncRNA SNHG6 knockdown inhibits cisplatin resistance and progression of gastric cancer through miR-1297/BCL-2 axis
Source: Biosci Rep. 2021 Dec 8;41(12):BSR20211885. doi: 10.1042/BSR20211885 (PMC8661508; doi:10.1042/BSR20211885)
Supplement: Supplementary Data [file BSR-2021-1885_supp1.zip › BSR-2021-1885_suppSM3.pptx]

## Slide 1
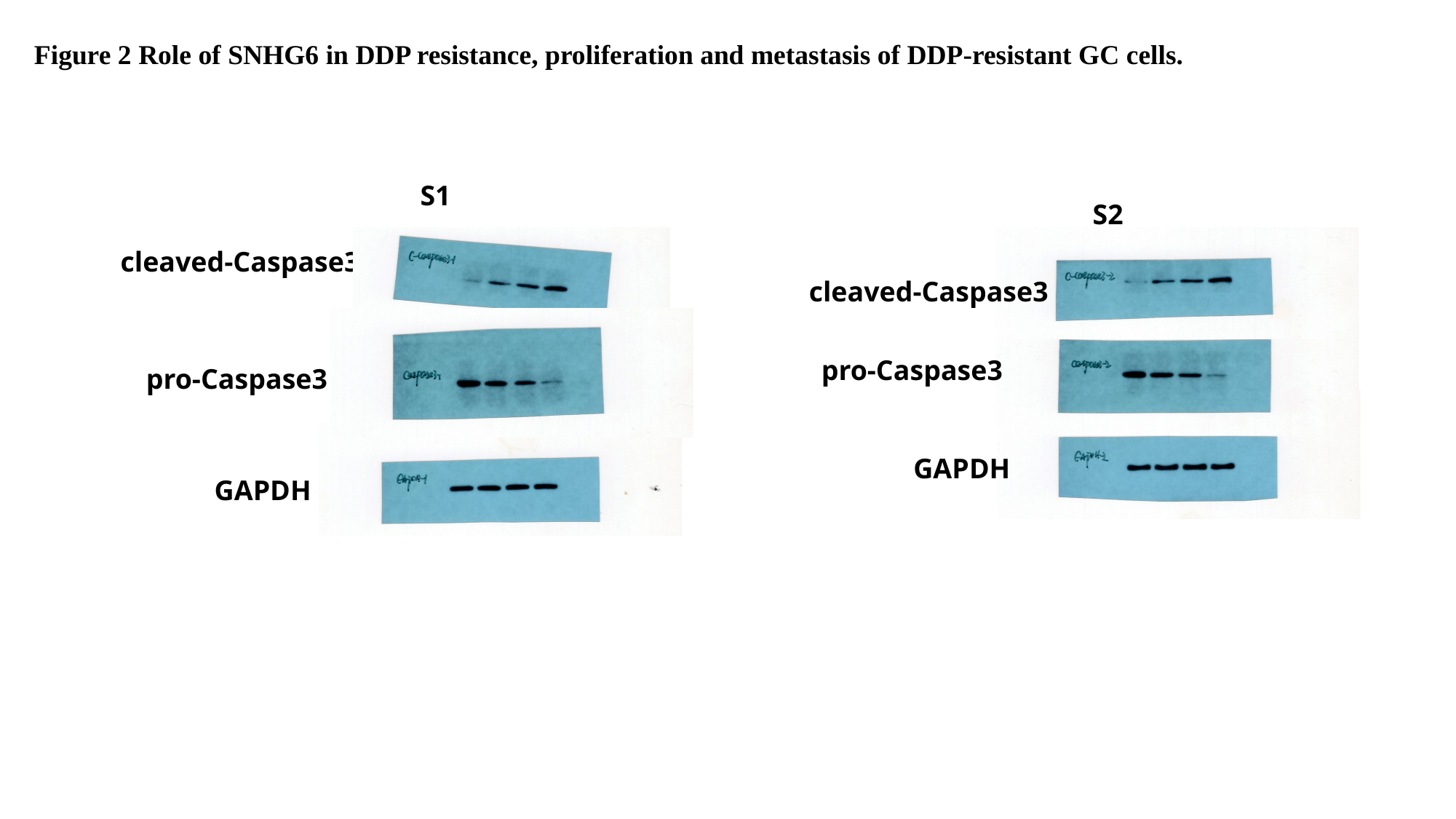

Figure 2 Role of SNHG6 in DDP resistance, proliferation and metastasis of DDP-resistant GC cells.
S1
S2
cleaved-Caspase3
cleaved-Caspase3
GAPDH
GAPDH
pro-Caspase3
pro-Caspase3

## Slide 2
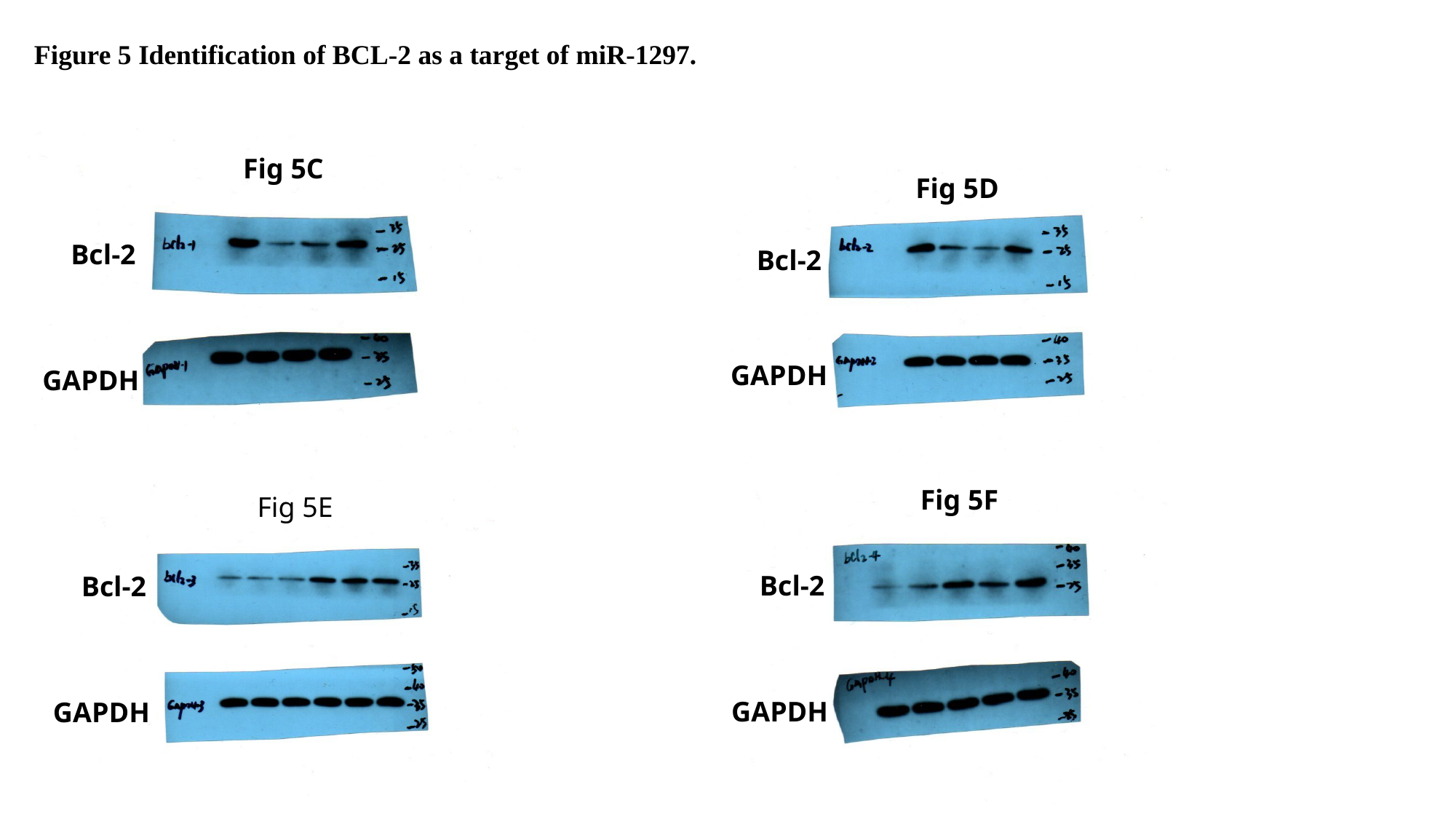

Figure 5 Identification of BCL-2 as a target of miR-1297.
Fig 5C
Fig 5D
Bcl-2
Bcl-2
GAPDH
GAPDH
Fig 5F
Fig 5E
Bcl-2
Bcl-2
GAPDH
GAPDH

## Slide 3
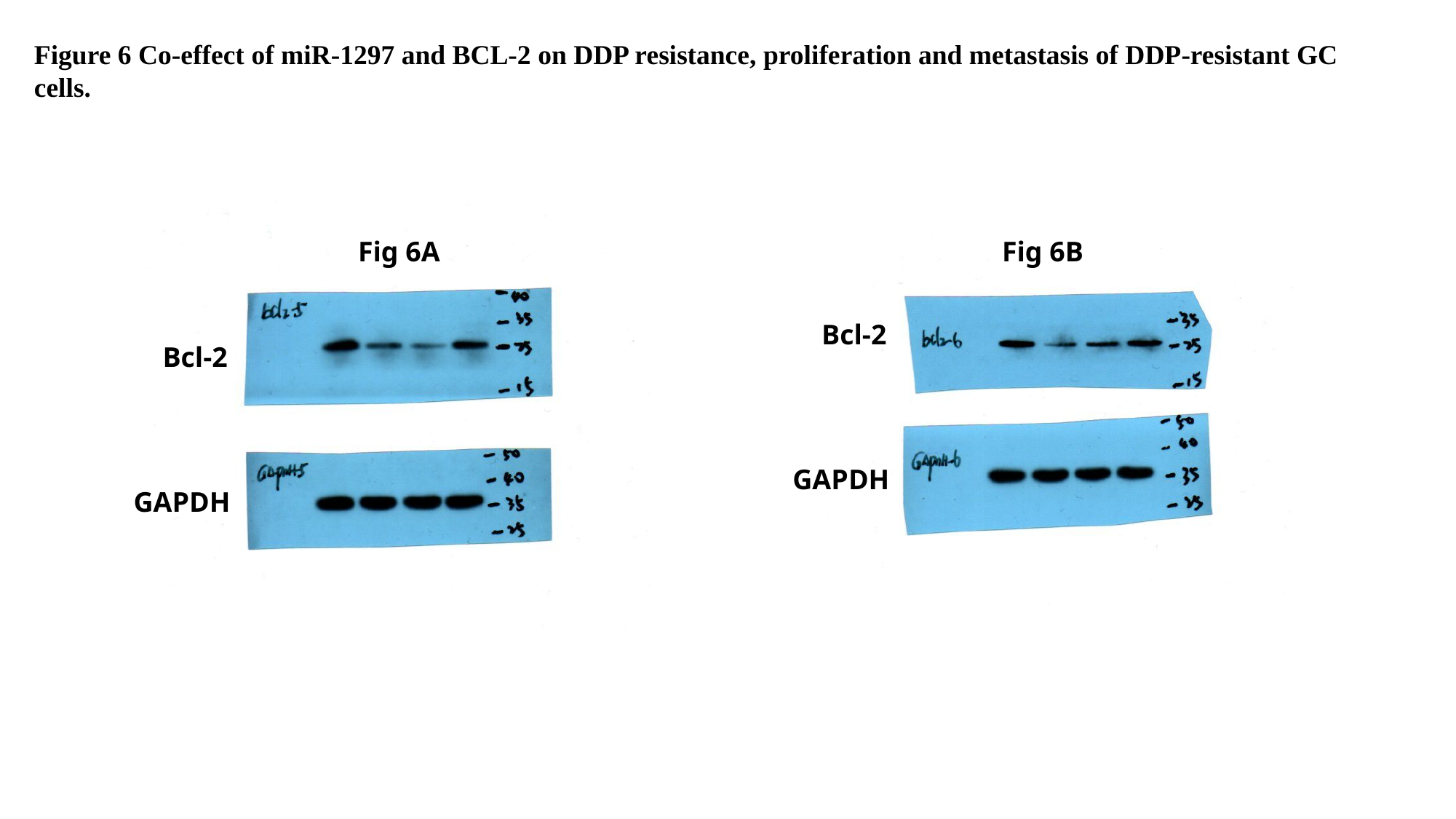

Figure 6 Co-effect of miR-1297 and BCL-2 on DDP resistance, proliferation and metastasis of DDP-resistant GC cells.
Fig 6A
Fig 6B
Bcl-2
Bcl-2
GAPDH
GAPDH
