# Supplementary figures and images for: LncRNA SNHG6 knockdown inhibits cisplatin resistance and progression of gastric cancer through miR-1297/BCL-2 axis
Source: Biosci Rep. 2021 Dec 8;41(12):BSR20211885. doi: 10.1042/BSR20211885 (PMC8661508; doi:10.1042/BSR20211885)

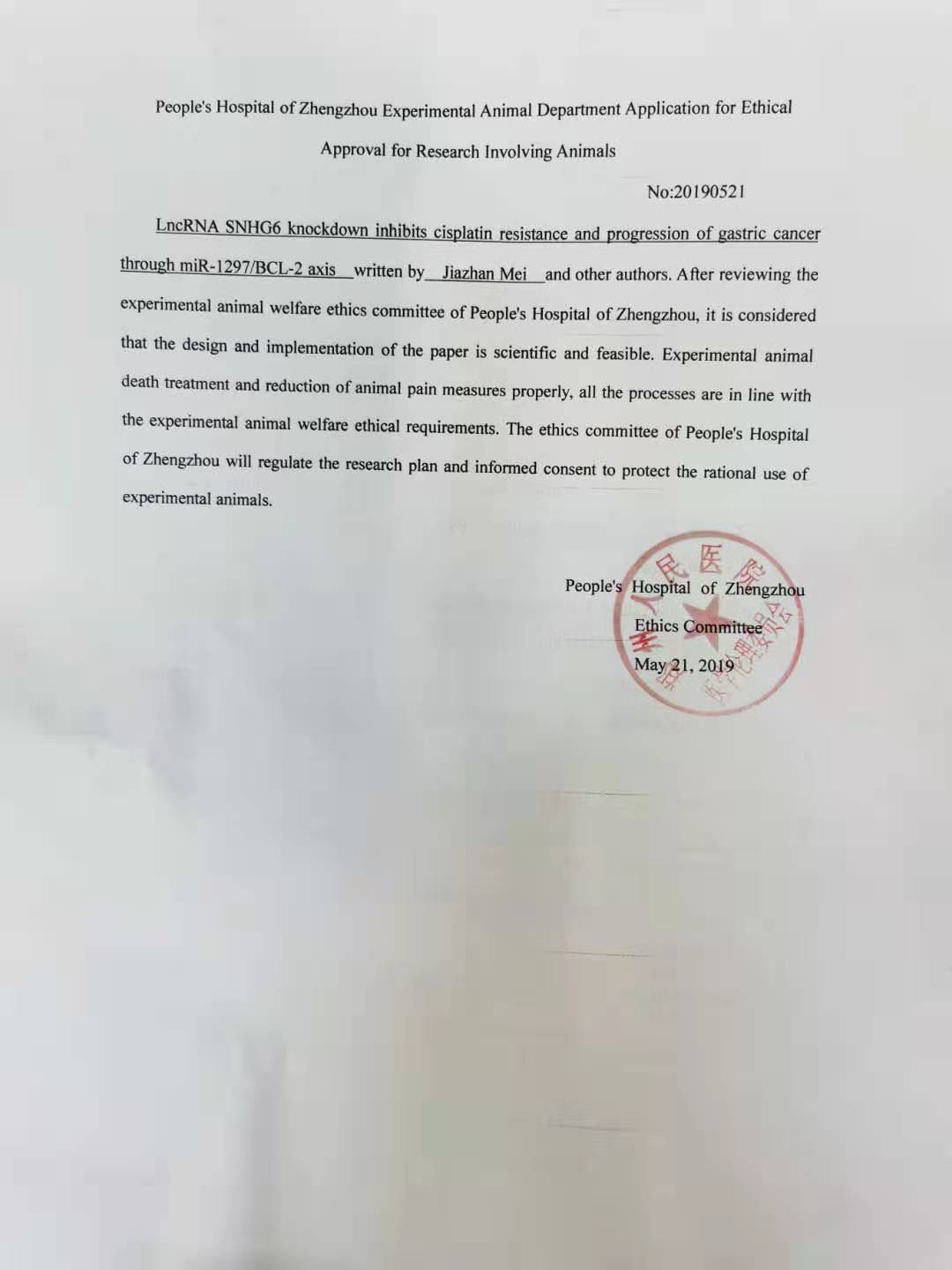

Supplement: Supplementary Data [file BSR-2021-1885_supp1.zip › BSR-2021-1885_suppSM1.jpg]

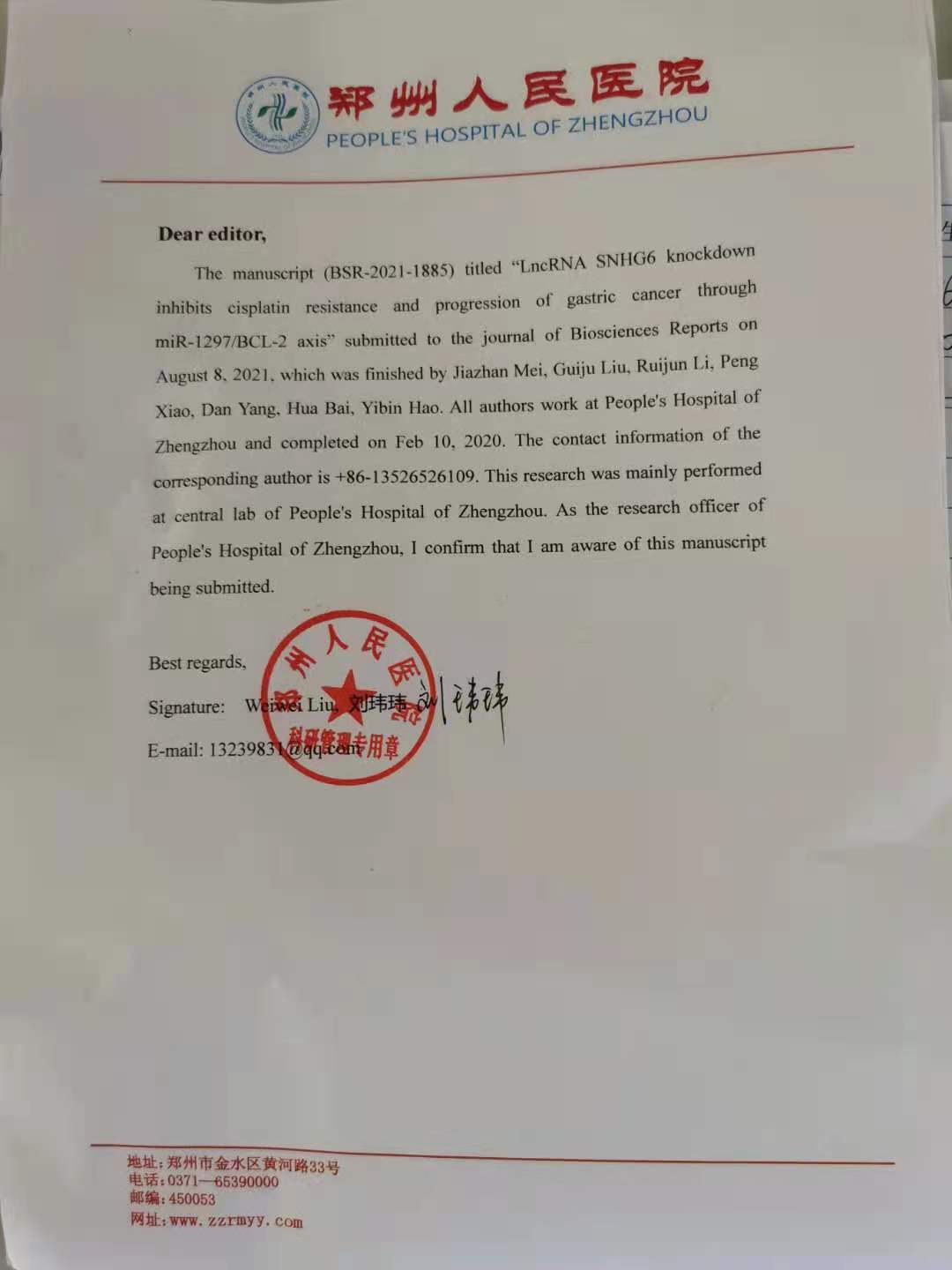

Supplement: Supplementary Data [file BSR-2021-1885_supp1.zip › BSR-2021-1885_suppSM2.jpg]

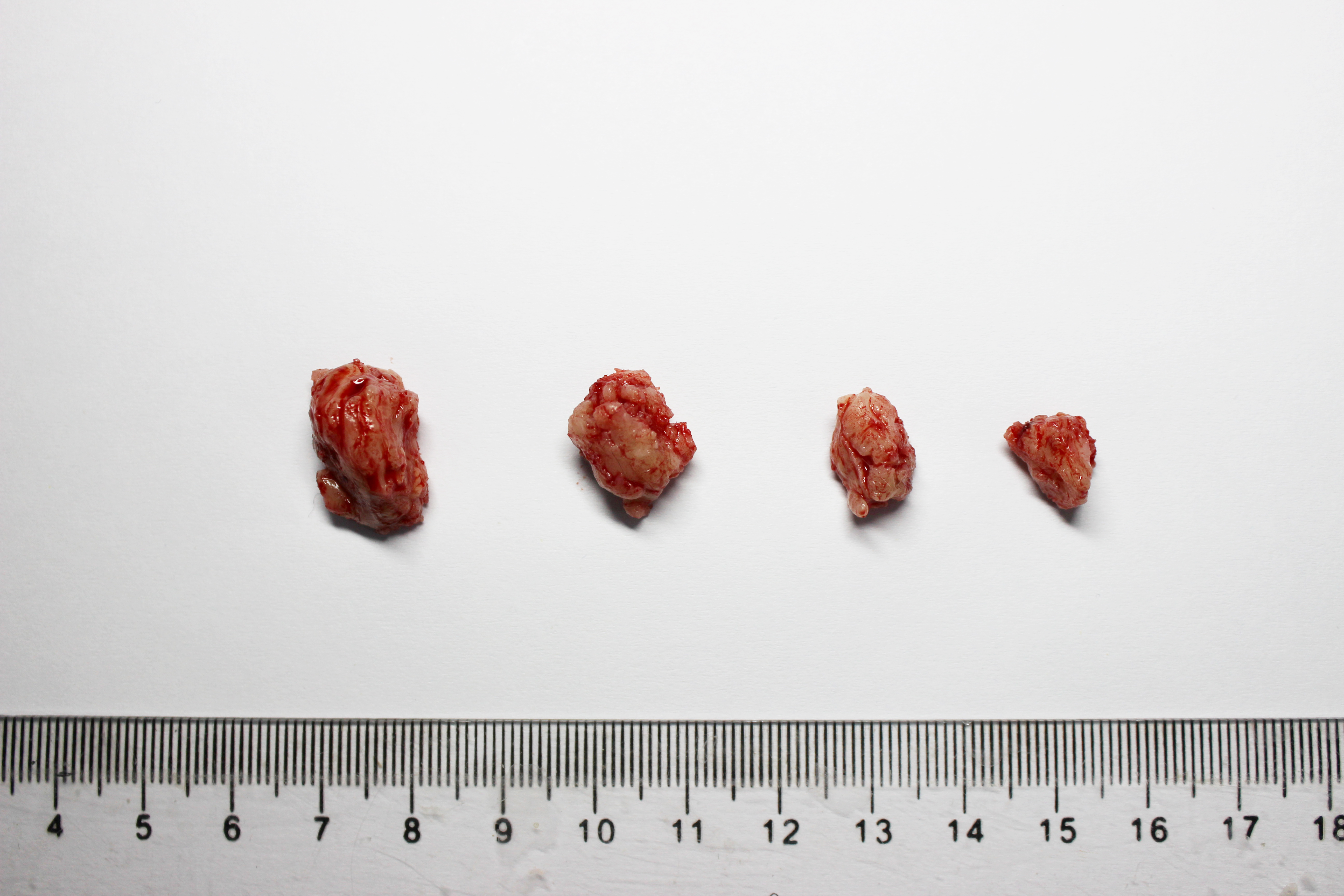

Supplement: Supplementary Data [file BSR-2021-1885_supp1.zip › BSR-2021-1885_suppSM4.jpg]
